# Reliability Estimation in Multidimensional Scales: Comparing the Bias of Six Estimators in Measures With a Bifactor Structure

**DOI:** 10.3389/fpsyg.2021.508287

**Published:** 2021-06-24

**Authors:** Italo Trizano-Hermosilla, José L. Gálvez-Nieto, Jesús M. Alvarado, José L. Saiz, Sonia Salvo-Garrido

**Affiliations:** ^1^Departamento de Psicología, Facultad de Educación, Ciencias Sociales y Humanidades, Universidad de La Frontera, Temuco, Chile; ^2^Departamento de Trabajo Social, Facultad de Educación, Ciencias Sociales y Humanidades, Universidad de La Frontera, Temuco, Chile; ^3^Departamento de Psicobiología y Metodología en Ciencias del Comportamiento, Facultad de Psicología, Universidad Complutense de Madrid, Madrid, Spain; ^4^Departamento de Matemática y Estadística, Facultad de Ingeniería y Ciencias, Universidad de La Frontera, Temuco, Chile

**Keywords:** reliability, multidimensional, bifactor, Monte – Carlo simulation, measurement

## Abstract

In the context of multidimensional structures, with the presence of a common factor and multiple specific or group factors, estimates of reliability require specific estimators. The use of classical procedures such as the alpha coefficient or omega total that ignore structural complexity are not appropriate, since they can lead to strongly biased estimates. Through a simulation study, the bias of six estimators of reliability in multidimensional measures was evaluated and compared. The study is complemented by an empirical illustration that exemplifies the procedure. Results showed that the estimators with the lowest bias in the estimation of the total reliability parameter are omega total, the two versions of greatest lower bound (GLB) and the alpha coefficient, which in turn are also those that produce the highest overestimation of the reliability of the general factor. Nevertheless, the most appropriate estimators, in that they produce less biased estimates of the reliability parameter of the general factor, are omega limit and omega hierarchical.

## Introduction

This article examines the biases that can occur when estimating the reliability of the total score of a multi-item measure when the latent structure of that set of items corresponds to a bifactor model. The majority of internal consistency reliability coefficients were deduced based on the assumption that the items were homogeneous in that they measure a single construct, that is, unidimensionality was assumed (Graham, 2006; Peters, 2014). For this reason, the current literature (Green and Yang, 2009, 2015; Crutzen and Peters, 2017) underlines the need to evaluate the factorial structure of constructs (their dimensionality) of multi-item measures, as a pre-exam step of its reliability.

Perfect unidimensionality, also known as strict unidimensionality, refers to the presence of a single common factor that adequately explains the inter-item covariance matrix (or correlations). However, perfect unidimensionality is a difficult requirement to meet (ten Berge and Sočan, 2004) since, in practice, there is usually inter-item covariance beyond the common factor, which suggests a certain degree of multidimensionality (Reise et al., 2000; Sočan, 2000). Strict unidimensionality requires that there be no specific groupings or factors and that there is no correlation between item errors, since these correlations indicate that the items share variance beyond that explained by the common factor. For example, the redundancy of the content of the items (asked more than once, but with slightly different wording) or similarity in the way the items are presented are usually indicated as sources of this additional variance, other than the variance due to the common factor. To the extent that additional sources of variance, such as that attributed to correlations between errors or to group or specific factors, allow complex structures to be modeled, estimates of the reliability of the common factor will require controlling those sources of additional variance.

When items with very similar content are included in a test (i.e., content overlap), a positive correlation between errors is observed. If this correlation between errors is not controlled in a unidimensional test, for example by applying the Raykov formula (2001), the alpha, total omega, or GLB reliability coefficients will overestimate the true reliability of that scale.

If instead of two items with similar content, there are three or more items with content overlap, new factors will be generated so that if their specific variance is not controlled by bifactor modeling (Reise, 2012) that allows hierarchical omega estimation, the alpha, total omega, and GLB estimations will overestimate reliability by ignoring the multidimensionality produced by correlated errors. Another example of a similar overestimation is observed in tests with overlapping alternatives, as it is the case of the testlet (Gessaroli and Folske, 2002; Teker and Dogan, 2015).

Considering the difficulty of obtaining strictly unidimensional measures, a more realistic alternative is to investigate the so-called essential unidimensionality, which implies the coexistence of a general factor, common to all items, and of group or specific factors that are only common to some subsets of items. The so-called bifactor model is the most recommended procedure (Reise, 2012; Rodriguez et al., 2016) to evaluate the essential unidimensionality of a multi-item measure.

When the strict unidimensionality assumption is violated, most estimators tend to produce biases that affect the correct estimate and interpretation of reliability (Raykov, 2001; ten Berge and Sočan, 2004; Green and Yang, 2015; Crutzen and Peters, 2017). Of particular relevance are the overestimation biases of true reliability (Revelle and Zinbarg, 2009; Yang and Green, 2011; Sijtsma, 2012; Dunn et al., 2014) since they generate the false sensation of being accurately measured when, in fact, that estimate is positively biased (Viladrich et al., 2017).

Cronbach’s alpha coefficient is the main estimator of reliability despite its limitations (Green and Yang, 2009; Sijtsma, 2009a, 2012; Yang and Green, 2011; Cho and Kim, 2015; Trizano-Hermosilla and Alvarado, 2016; McNeish, 2018). In bifactor structures, the alpha coefficient usually overestimates the reliability of the general or common factor (Zinbarg et al., 2006) and underestimates the reliability attributed to all the factors of the model, including simultaneously the common and specific factors (Revelle and Zinbarg, 2009). In fact, Cortina (1993) recommends not using this coefficient in multidimensional measurements, arguing that if the set of items in a test is explained by orthogonal (or poorly correlated) factors, the alpha coefficient of that set of items can provide values greater than 0.70 when the true reliability is much lower and even nil in balanced structures in which orthogonal factors provide equal variance. Even in cases where there are correlations between errors, the alpha coefficient delivers higher reliability values than would be expected if the relevant corrections were made, as long as the correlations between the errors of these items are positive (Raykov, 2001). The Cronbach’s alpha coefficient is equivalent to the lambda 3 proposed by Guttman (1945). The other lambda estimators, as well as alpha, do not take into account the possible multidimensionality of the measure. Given these limitations, it is necessary to examine whether other estimators, other than alpha, turn out to be more suitable for computing the reliability of multidimensional measurements. This could be the case of the omega coefficient family (Zinbarg et al., 2006; Revelle and Zinbarg, 2009; Green and Yang, 2015).

One of the alternatives to the traditional alpha coefficient is the family of omega coefficients (McDonald, 1999; Zinbarg et al., 2005, 2006). Of these, the best known are the total omega and the hierarchical omega coefficient. Total omega is used to evaluate the joint reliability of all the factors of the model, without differentiating between the sources of variance of specific or general factors, while the hierarchical omega coefficient is used to correctly estimate the reliability of the general factor, controlling the variance of the specific factors (Green and Yang, 2015). The main advantages of these coefficients are that they are evaluated within a factorial model, have more realistic assumptions than the alpha coefficient (Dunn et al., 2014) and allow clarifying the distinctions between validity and reliability (Green and Yang, 2009).

Despite GLB’s solid theoretical foundation (Sijtsma, 2009a) it has not been used often by researchers (Sijtsma, 2009b), although some recent empirical studies have shown that it behaves better than alpha (Lila et al., 2014) and is less biased in unidimensional scales than alpha and omega (Wilcox et al., 2014; Trizano-Hermosilla and Alvarado, 2016). However, in samples of less than 1000 cases and under the assumption of normality, it has been observed that this coefficient tends to overestimate the true value of reliability (Shapiro and ten Berge, 2000). Revelle and Zinbarg (2009) argue that GLB, contrary to its name, is not the greatest lower limit of reliability, but that other coefficients, such as total omega, allow for clearer estimations of reliability and in some cases obtain higher values than those presented by GLB. Beyond the debate about which estimator to choose in strictly unidimensional structures, it is necessary to conduct simulation studies to determine which is the best reliability alternative when the assumption of unidimensionality is questionable in terms of goodness of fit, but when a strong general common factor is observed according to the theoretical model (i.e., bifactor model).

A simulation study assessed and compared bias in six estimators of reliability in bifactor models. In addition to the Cronbach’s alpha coefficient, three Omega coefficients (Hierarchical, Total, and Limit) and two versions of the greatest lower bound coefficient (GLBFa and GLBAlgebraic) were examined. In addition, for illustrative purposes, these coefficients were evaluated and compared using real data.

## Methods

### Data Conditions

**FIGURE 1:**
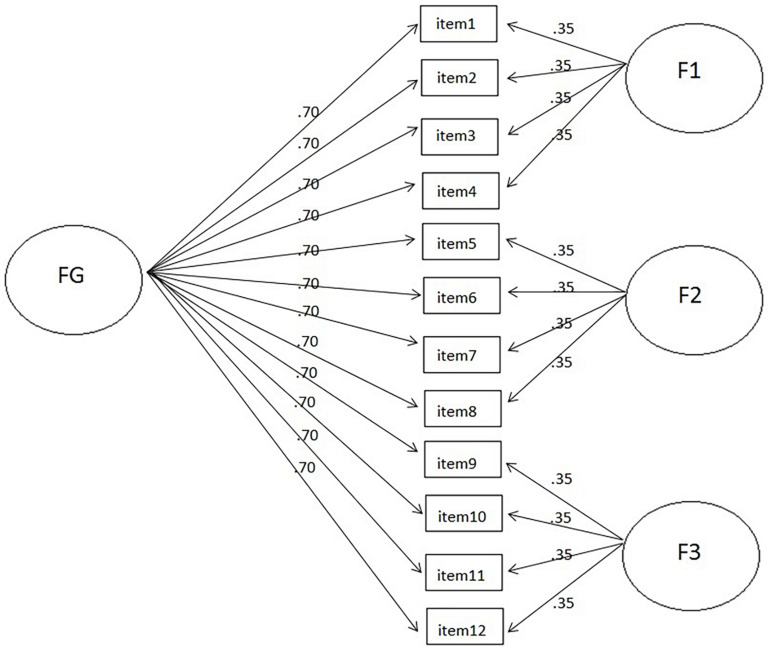
Example of a 12-item bifactor model, with one general factor, with loadings = 0.70 and three specific factors, with loadings = 0.35.

Eighty-one bifactor models with six sample sizes were simulated, manipulating four independent variables: (1) nine loadings sizes for the general factor, with values of 0.40, 0.45, 0.50, 0.55, 0.60, 0.65, 0.70, 0.75, and 0.80; (2) three loading sizes for the specific factors, with values of 0.35, 0.45, and 0.55; (3) six sample sizes of 100, 150, 200, 250, 500, and 1,000; and (4) three test lengths with 12, 24, and 48 items, thus considering a wide range of conditions that can be observed in real situations. The simultaneous consideration of these four conditions generated a total of 486 specific combinations, with 500 replications being made per condition. An example of one of these conditions is shown in Figure 1, which includes 12 items organized according to a bifactor structure composed by a well-defined general factor with high saturation (loading = 0.70), as well as three poorly defined specific factors with low saturation (loading = 0.35).

### Analysis of Data

As dependent variables, the bias [Σ ($\hat{\rho }$-*ρ*)/*Nr*]-where $\hat{\rho }$ represents the estimator, *ρ* the true reliability, and *Nr* the number of replications- of each of the six coefficients was obtained regarding both the reliability attributable to the general or common factor (general reliability) and that attributable to all common and specific factors of the model (total reliability). Bias levels close to zero were indicative of an adequate estimate of the parameter. Then, descriptive analyses of the center and dispersion statistics of the global bias of each estimator were performed. Next, by calculating the coefficient of determination (*r*^2^, understood as the squared correlation between the true reliability and each of the reliability estimators), the percentage of variance shared between each reliability estimator and the parameter of true reliability was examined, both for general reliability and for total reliability, for each condition, presenting the mean between conditions. A high *r*^2^ value was indicative of greater overlap between the estimator and true reliability, suggesting a less biased estimator. Subsequently, the behavior of the six reliability estimators was evaluated using data collected through the Authoritative School Climate Scale (ASCS; Cornell and Huang, 2016) in 1,868 Chilean adolescent students. This scale includes 15 items grouped into two highly correlated dimensions. With these data and by using confirmatory factor analysis (CFA), three models were examined first: a unidimensional, a bidimensional and a bifactor model. Four measures of goodness of fit (Chi-square, CFI, TLI, and RMSEA) were obtained and their values were interpreted according to the acceptance criteria conventionally established in the literature (Schreiber et al., 2006). In addition, the Explained Common Variance (ECV) was estimated, which is the common variance explained by the general factor divided by the total common variance, indicating the degree of unidimensionality, or relative strength, of general to group factors (Reise et al., 2013; Rodriguez et al., 2016). Factorial analyses with empirical data were performed with Mplus 8.1 software (Muthén and Muthén, 1998–2017) while reliability estimates were obtained using the psych package (Revelle, 2020) in R software (R Core Team, 2016).

## Results

The results shown below were obtained through all conditions.

### Coefficient Bias When Estimating the Reliability of the General Factor and Total Reliability

Table 1 shows the global descriptive statistics of the bias levels for each of the six coefficients. When considering the reliability of the general factor as a parameter, the Omega Limit coefficient, which corresponds to an asymptotic version of Omega Hierarchical, presents the average bias closest to zero. This result indicates that Omega Limit tends to deliver less biased estimates of general factor reliability. The Omega Hierarchical coefficient also exhibits a small average bias, close to zero, although negative. The remaining four coefficients (Omega Total, Cronbach’s Alpha, GLBFa, and GLBAlgebraic) have positive averages bias, indicating that they tend to overestimate the reliability of the overall factor. Regarding the dispersion of the estimators, it was observed that Omega Limit and Omega Hierarchical presented the lowest dispersion values for the reliability of the general factor. On the contrary, Omega Total and GLBFa estimators presented the lowest SD in the estimation of total reliability. In the Supplementary Material, two graphs display the behavior of these coefficients as they are used to estimate the reliability of the general factor as a function of the different manipulated factors.

**TABLE 1:** Overall descriptive statistics of the level of bias when estimating the General reliability and total reliability of each coefficient.

Parameter	Estimator	Average	SD	Minimum	Maximum
General factor reliability	Omega hierarchical	–0.039	0.047	–0.459	0.135
	Omega limit	0.001	0.046	–0.393	0.293
	Omega total	0.148	0.071	–0.036	0.386
	Cronbach’s alpha	0.128	0.065	–0.206	0.375
	GLBFa	0.153	0.072	0.030	0.397
	GLBAlgebraic	0.164	0.077	–0.036	0.630
Total reliability	Omega hierarchical	–0.186	0.085	–0.731	–0.033
	Omega limit	–0.146	0.079	–0.716	0.119
	Omega total	0.001	0.008	–0.226	0.103
	Cronbach’s alpha	–0.019	0.017	–0.446	0.053
	GLBFa	0.007	0.012	–0.075	0.138
	GLBAlgebraic	0.017	0.015	–0.120	0.300

*GLBFa, greatest lower bound by factorial methods; GLBAlgebraic, greatest lower bound using algebraic methods.*

When evaluating the degree of bias presented by the coefficients when estimating the reliability of all the factors, it can be seen that Omega Total and GLBFa show the levels closest to 0. The former presents the least variability, indicating that it tends to provide unbiased estimates of the reliability of all factors, while GLBAlgebraic has a slight positive bias, and the Alpha coefficient shows a slight negative bias when estimating the reliability of all factors. It is observed that Omega Hierarchical and Omega Limit present considerable negative biases when estimating the reliability of all factors.

### Coefficients of Determination Between Estimators and True Reliability

As observed in Table 2, the Omega Hierarchical and Omega Limit coefficients share 84.5% and 78.7% of variance, respectively, with the true reliability attributed to the general factor (the two with the least bias and variability). In the other coefficients, the percentages of shared variance are lower and range from 54.9% (Alpha) to 40.8% (GLBAlgebraic).

**TABLE 2:** Determination coefficients (*r*^2^) between the reliability estimators and the parameters of general reliability and total reliability.

	Omega hierarchical	Omega limit	Omega total	Cronbach’s alpha	GLBFa	GLBAlgebraic
General factor reliability	0.845**	0.787**	0.500**	0.549**	0.498**	0.408**
Total reliability	0.569**	0.326**	0.974**	0.941**	0.945**	0.924**

GLBFa, greatest lower bound by factorial methods; GLBAlgebraic, greatest lower bound using algebraic methods. **p < 0.001.

Meanwhile, when evaluating the relationship between the coefficients of reliability and the reliability attributable to all the factors of the model (total reliability), it is observed that Omega Total has the highest coefficient of determination, followed closely by GLBFa, then by Cronbach’s alpha and finally, by GLBAlgebraic. The Omega Total and GLBFa estimators presented the least bias and the least dispersion in the descriptive analyses. It can also be seen that Omega, in its Hierarchical and Limit version, has a comparatively low association with total reliability (see Table 2).

### An Illustration With Real Data

A CFA of the responses to the ASCS provided unsatisfactory adjustment rates for the unidimensional model, χ^2^ (90) = 2,271.382, *p* < 0.001, CFI = 0.937, TLI = 0.927, RMSEA = 0.111 (90% CI 0.107–0.115). However, these indices were relatively acceptable for the bidimensional model χ^2^ (89) = 1,704.840, *p* < 0.001, CFI = 0.954, TLI = 0.945, RMSEA = 0.096 (90% CI 0.092–0.100), with a correlation between both factors of 0.89 and significantly better values for the bifactor model χ^2^ (75) = 1,062.371, *p* < 0.001, CFI = 0.972, TLI = 0.960, RMSEA = 0.082 (90% CI 0.078–0.086). Considering these fit values, it was decided to analyze the reliability of the bifactor model. For the general factor, the estimated reliability was 0.785 (Omega Hierarchical), 0.861 (Omega Limit), 0.912 (Omega Total), 0.900 (Alpha and GLBFa), and 0.931 (GLBAlgebraic). The ECV was 0.795, suggesting the existence of a strong common factor. As expected, the Omega Hierarchical and Omega Limit estimators obtained comparatively lower reliability values, giving a less biased estimate of reliability since these coefficients control for the variance from the specific factors. On the other hand, Alpha, Omega Total, and the two GLB versions obtained estimates higher than 0.90 when the total reliability was examined. As these four coefficients do not distinguish between the different sources of variance, it can be said that they have a positive bias in estimating the reliability of the general factor in bifactor measurements.

## Discussion

In this simulation study, which was illustrated by an analysis of real data, the behavior of six reliability estimators was evaluated when applied to latent multidimensional bifactor structures. While the literature clearly points out the advantages and disadvantages of each of these coefficients (Zinbarg et al., 2005, 2006; Revelle and Zinbarg, 2009; Sijtsma, 2009a; Yang and Green, 2011; Cho and Kim, 2015; Trizano-Hermosilla and Alvarado, 2016; McNeish, 2018), the practical implications are usually poorly understood by applied researchers (Sijtsma, 2012; Sijtsma and van der Ark, 2015).

In this study, we examined the reliability of the general factor in bifactor models (Zinbarg et al., 2005, 2006; Reise, 2012), a situation in which the most suitable coefficients proved to be Omega Hierarchical and Omega Limit due to their low bias and low dispersion. The Omega Total, Alpha, and the two versions of GLB show levels of overestimation biases of that true reliability, delivering inflated values since they do not distinguish between the variances attributed to the general factor and the specific factors. Thus, these four estimators are not recommended to examine the reliability of the general factor in bifactor models, except when the loadings of the general factor are high, and the loadings of the specific factors are very small (see Supplementary Material). In a recent work by Green and Yang (2015) on reliability in the bifactor model, the proper functioning of Omega Hierarchical has been observed.

When estimating the reliability of all factors (both general and specific), the most accurate estimators of the population parameter in order are: the Omega Total coefficient, the two versions of GLB and third, the Alpha coefficient, since the latter presented a slight negative bias when estimating the parameter. Recently, Raykov (2019) showed that Alpha is a good population estimator, which is consistent with our results, although the study did not analyze the behavior of other estimators. According to our results, Omega Total (Revelle and Zinbarg, 2009) and GLBFa (Sijtsma, 2009a) are the estimators that have the greatest association with the total reliability. This apparently favorable result hides a strong overestimation of the reliability of the general factor. Therefore, the Omega Total and GLB coefficients should not be used in bifactor structures to determine the precision with which the general attribute of interest is measured. As indicated by Trizano-Hermosilla and Alvarado (2016), GLB is deduced from the assumption that there is no correlation between errors, so that in conditions of strict unidimensionality their behavior is excellent. However, when this assumption is generally unfulfilled, even when the scale was initially designed to be unidimensional, the correlation between errors becomes part of the true variance and consequently there is a strong overestimation of the general factor, as with Omega Total and to a lesser extent the Alpha coefficient.

The results obtained with real data illustrate the findings of the previous simulation study. The first step in any practical application should be to show the goodness of fit to the bifactor model (Green and Yang, 2015). Among the six coefficients examined, Omega Hierarchical and Omega Limit delivered comparatively less biased values when estimating the reliability of the general factor in bifactor models. On the other hand, the coefficients Alpha, Omega Total, and the two versions of GLB, being exposed to a positive biased estimate of the reliability of the general factor, provide higher values of reliability than such a general factor really has.

Regarding the limitations of this article, it should be mentioned that the estimator bias results were presented without detailed evaluations between the conditions and their interactions due to the low dispersion of the Omega Limit and Omega Hierarchical estimators. However, the high dispersion shown by the unidimensional estimators indicates that their values are sensitive to the specific conditions of application and therefore, before using them, additional simulations should be conducted to find their magnitude of bias in the specific scenario. Future lines of research could expand upon the findings of the present study by considering categorical variables in the simulation.

We invite researchers and editors to use the current recommendations regarding the correct estimation of the internal structure of multi-item measures, and then proceed to examine the evidence of reliability by means of the coefficients that offer less bias in the parameter estimation. Obtaining unbiased values of the reliability with which a certain attribute is being measured allows avoiding the acceptance of measures that show high total reliability, without differentiating between general and specific factors, due to positively biased estimates. The relevance of this advice is that these biased estimates affect the quality of inferences that can be made from the scores obtained from these measures.

## Data Availability Statement

The dataset generated for this study are available on request to the corresponding author.

## Author Contributions

IT-H contributed to general direction of the article, writing analysis and interpretation, as well as in the generation of simulation work. JLG-N contributed to the empirical aspects of the analysis and writing. JMA contributed specifically to the design of the article and simulation conditions as well as to the interpretation of the results, also made a critical evaluation of the article. JLS and SS-G performed a global critical review of the article and improving it specifically. All authors contributed to the article and approved the submitted version.

## Conflict of Interest

The authors declare that the research was conducted in the absence of any commercial or financial relationships that could be construed as a potential conflict of interest.
