# Supplementary material for: Reliability Estimation in Multidimensional Scales: Comparing the Bias of Six Estimators in Measures With a Bifactor Structure
Source: Front Psychol. 2021 Jun 24;12:508287. doi: 10.3389/fpsyg.2021.508287 (PMC8263896; doi:10.3389/fpsyg.2021.508287)

Supplementary material

Two sets of graphs describing the level of bias obtained for each of the estimators based on the different conditions evaluated in this study are presented below.

The first set of graphs shows the level of bias (Y axis) as a function of the number of items (12, 24 and 48), the sample size (100, 150, 200, 250, 500 and 1000) and the factorial loadings of the general factor (.40, .45, .50, .55, .60, .65, .70, .75, .80). As the size of the factorial loading of the general factor increases, less bias is observed in all the coefficients. Additionally, better Omega Hierarchical performance is observed with samples of 500 or more, while better Omega Limit performance is observed with smaller samples, with smaller overall factor saturations and fewer items.

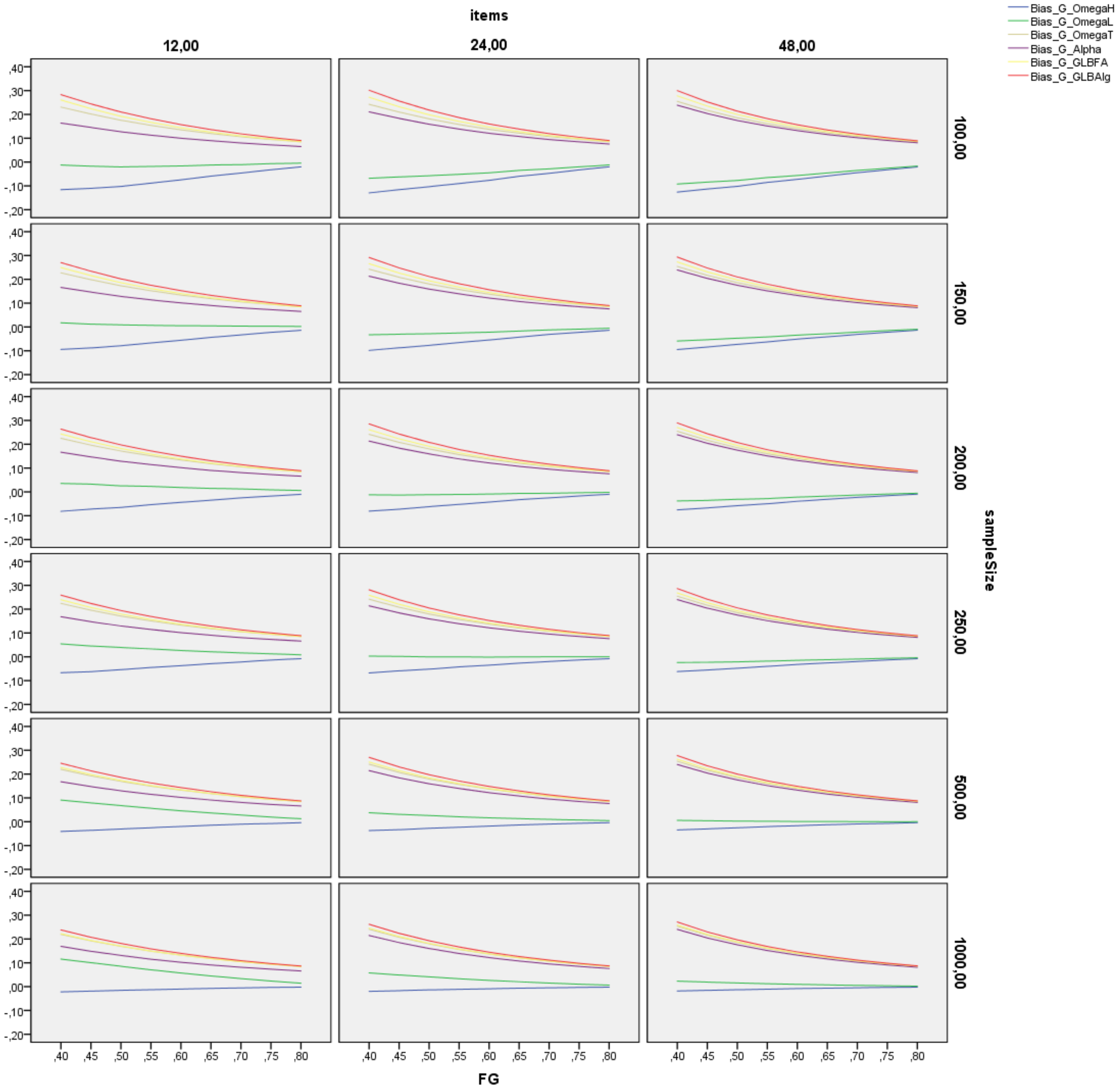

The second graph shows the level of bias (Y axis) as a function of the size of the factorial loadings of the specific factors (.35, .45, .55), the sample size (100, 150, 200, 250, 500 and 1000) and the factorial loadings of the general factor (.40, .45, .50, .55, .60, .65, .70, .75, .80). It can be seen that with low loadings in the specific factors and high loadings in the general factor, most of the estimators tend to converge with biases close to 0 (see first column and last three rows). This is due to conditions which reflect a strong general factor with well-defined loadings and weak specific factors (with loadings of .35). Meanwhile, as the size of the loadings of the specific factors increases, the discrepancies between the estimators will increase, generating strong positive biases for the alpha, total omega, GLBFA and GLBalgebraic estimators.

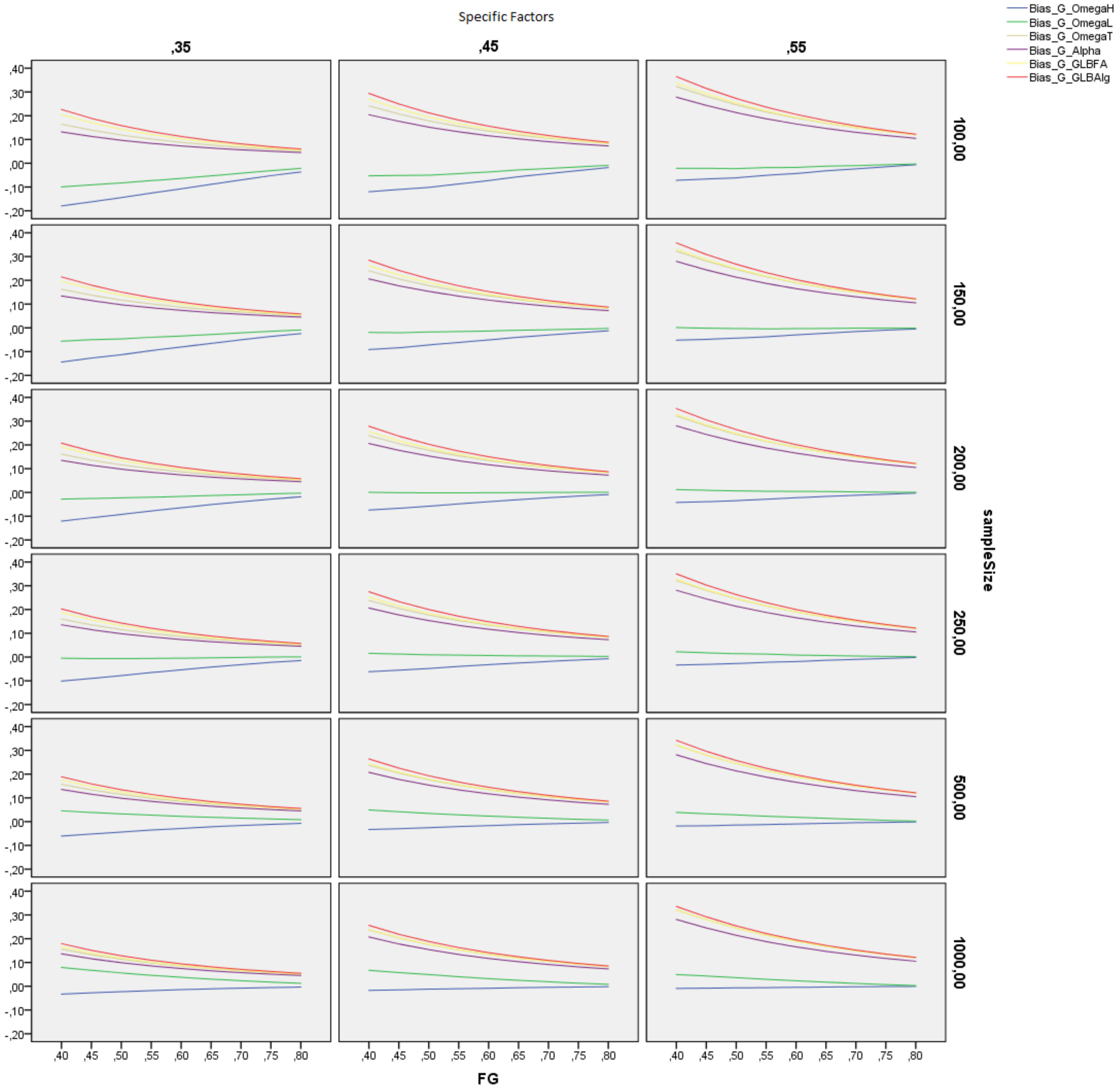

Supplement: Supplementary file 1 [file Data_Sheet_1.PDF]
